# Supplementary material for: Identification of Torquetenovirus Species in Patients with Kawasaki Disease Using a Newly Developed Species-Specific PCR Method
Source: Int J Mol Sci. 2023 May 12;24(10):8674. doi: 10.3390/ijms24108674 (PMC10218515; doi:10.3390/ijms24108674)
Supplement: Supplementary file 1 [file ijms-24-08674-s001.zip › ijms-2344996-supplementary.pdf]

Supplementary Table S1. The result from our ssTTV PCR applied to 33 individuals.

	Total			TTV1			TTV5			TTV7			TTV13			TTV15			TTV22			TTV24			TTV28†			TTV29†		
seq	K	D	R	K	D	R	K	D	R	K	D	R	K	D	R	K	D	R	K	D	R	K	D	R	K	D	R	K	D	R
1	6.9	6.4	5	32			28			9.2				22		30	40				30	21	20					Pos		
2	1.6	1.8																												
3																														
4	4.5	2.5	5													37	29													
5	6.2	4.8	2.5							25																				
6	3.8	4	3.7					34	24							33							38							
7	4.4	4.6	4.8					42	20													29	19							
8	2.6	6.1	5.1						27					21			27						30							
9	2		2.8															36												
10			1.3																		39									
11	4.7	5.1	3																	40										

† Qualitative PCR was applied to TTV28 and TTV29. Quantitative PCR was applied to other TTV species. Viral quantity is expressed by the threshold cycle which is inversely related to the viral load.

K: Kawasaki Disease. D: Diarrhoeal control group. R: Respiratory Infection control group.
